# High School Quality and 56-Year All-Cause Mortality Risk Across Race and Ethnicity

**DOI:** 10.1093/geroni/igaa057.1632

**Published:** 2020-12-16

**Authors:** Dominika Seblova, Kelly Peters, Susan Lapham, Laura Zahodne, Tara Gruenewald, Maria Glymour, Benjamin Chapman, Jennifer Manly

**Affiliations:** 1 Columbia University Medical Center, New York, New York, United States; 2 American Institutes for Research, Washington, District of Columbia, United States; 3 University of Michigan, Ann Arbor, Michigan, United States; 4 Chapman University, Orange, California, United States; 5 University of California, San Francisco, California, United States; 6 University of Rochester Medical Center, Rochester, New York, United States; 7 Columbia University, New York, New York, United States

## Abstract

Having more years of education is independently associated with lower mortality, but it is unclear whether other attributes of schooling matter. We examined the association of high school quality and all-cause mortality across race/ethnicity. In 1960, about 5% of US high schools participated in Project Talent (PT), which collected information about students and their schools. Over 21,000 PT respondents were followed for mortality into their eighth decade of life using the National Death Index. A school quality factor, capturing term length, class size, and teacher qualifications, was used as the main predictor. First, we estimated overall and sex-stratified Cox proportional hazards models with standard errors clustered at the school level, adjusting for age, sex, composite measure of parental socioeconomic status, and 1960 cognitive ability. Second, we added an interaction between school quality and race/ethnicity. Among this diverse cohort (60% non-Hispanic Whites, 23% non-Hispanic Blacks, 7% Hispanics, 10% classified as another race/s) there were 3,476 deaths (16.5%). School quality was highest for Hispanic respondents and lowest for non-Hispanic Blacks. Non-Hispanic Blacks also had the highest mortality risk. In the whole sample, school quality was not associated with mortality risk. However, higher school quality was associated with lower mortality among those classified as another race/s (HR 0.75, 95% CI: 0.56-0.99). For non-Hispanic Blacks and Whites, the HR point estimates were unreliable, but suggest that higher school quality is associated with increased mortality. Future work will disentangle these differences in association of school quality across race/ethnicity and examine cause-specific mortality.

